# Immune changes in hilar tumor draining lymph nodes following node sparing neoadjuvant chemoradiotherapy of localized cN0 non-small cell lung cancer

**DOI:** 10.3389/fonc.2023.1269166

**Published:** 2023-11-22

**Authors:** Jonathan Khalifa, Noémie Thébault, Clara-Maria Scarlata, Emma Norkowski, Carole Massabeau, Laurent Brouchet, Sophie Peries Bataille, Christelle Casaroli, Liza Vaz, Carine Valle, Emeline Sarot, Nathalie Saint-Laurent, Etienne Martin, Pierre-Benoît Pages, Alice Millière, Julien Mazières, Elizabeth Cohen-Jonathan Moyal, Françoise Lauzéral-Vizcaïno, Maha Ayyoub

**Affiliations:** ^1^ Université Toulouse III – Paul Sabatier, Inserm, CNRS, U1037, Université de Toulouse, Centre de Recherches en Cancérologie de Toulouse, Toulouse, France; ^2^ Department of Radiation Oncology, Institut Universitaire du Cancer de Toulouse-Oncopole, Institut Claudius Regaud, Toulouse, France; ^3^ Institut Universitaire du Cancer de Toulouse-Oncopole, Institut Claudius Regaud, Toulouse, France; ^4^ Department of Pathology, Institut Universitaire du Cancer de Toulouse-Oncopole, Institut Claudius Regaud, Toulouse, France; ^5^ Department of Thoracic Surgery, Centre Hospitalier Universitaire de Toulouse, Hôpital Larrey, Toulouse, France; ^6^ Centre de Ressources Biologiques – Cancer, Institut Universitaire du Cancer de Toulouse-Oncopole, Institut Claudius Regaud, Toulouse, France; ^7^ Department of Radiation Oncology, Centre Georges-François Leclerc, Dijon, France; ^8^ Department of Thoracic Surgery, Centre Hospitalo-Universitaire de Dijon, Dijon, France; ^9^ Department of Pathology, Centre Hospitalo-Universitaire de Dijon, Dijon, France; ^10^ Department of Thoracic Oncology, Centre Hospitalier Universitaire de Toulouse, Hôpital Larrey, Toulouse, France

**Keywords:** radiotherapy, tumor draining lymph node (TDLN), immune changes, non small cell lung cancer (NSCLC), lymph nodes sparing irradiation

## Abstract

**Background:**

While much progress has been accomplished in the understanding of radiation-induced immune effects in tumors, little is known regarding the mechanisms involved at the tumor draining lymph node (TDLN) level. The objective of this retrospective study was to assess the immune and biological changes arising in non-involved TDLNs upon node sparing concurrent chemoradiotherapy (CRT) of non-small cell lung cancer (NSCLC) tumors.

**Methods:**

Patients with proven localized (cN0M0) NSCLC, treated by radical surgery plus lymph node dissection with (CRT^+^) or without (CRT^-^) neoadjuvant chemoradiotherapy, whereby radiotherapy was targeted on the primary tumor with no significant incidental irradiation of the non-involved TDLN station (stations XI), were identified. Bulk RNA sequencing of TDLNs was performed and data were analyzed based on differential gene expression (DGE) and gene sets enrichment.

**Results:**

Sixteen patients were included and 25 TDLNs were analyzed: 6 patients in the CRT^+^ group (12 samples) and 10 patients in the CRT^-^ group (13 samples). Overall, 1001 genes were differentially expressed between the two groups (CRT^+^ and CRT^-^). Analysis with g-profiler revealed that gene sets associated with antitumor immune response, inflammatory response, hypoxia, angiogenesis, epithelial mesenchymal transition and extra-cellular matrix remodeling were enriched in the CRT^+^ group, whereas only gene sets associated with B cells and B-cell receptor signaling were enriched in the CRT^-^ group. Unsupervised dimensionality reduction identified two clusters of TDLNs from CRT^+^ patients, of which one cluster (cluster 1) exhibited higher expression of pathways identified as enriched in the overall CRT^+^ group in comparison to the CRT^-^ group. In CRT^+^ cluster 1, 3 out of 3 patients had pathological complete response (pCR) or major pathological response (MPR) to neoadjuvant CRT, whereas only 1 out of 3 patients in the other CRT^+^ cluster (cluster 2) experienced MPR and none exhibited pCR.

**Conclusion:**

Neoadjuvant node sparing concurrent CRT of NSCLC patients is associated with distinct microenvironment and immunological patterns in non-involved TDLNs as compared to non-involved TDLNs from patients with non-irradiated tumors. Our data are in line with studies showing superiority of lymph node sparing irradiation of the primary tumor in the induction of antitumor immunity.

## Introduction

For decades, radiotherapy has been shown to exert both local and systemic (abscopal) anti-tumor effects (1, 2). It was only in the early 2000’s that the systemic effects have been clearly attributed to antitumor immunity (3). Since then, major efforts have been deployed to decipher the mechanisms underlying what is classically called the *in situ* vaccination effect of radiotherapy.

Radiotherapy has the potency to release damage-associated molecular patterns (DAMPs) such as cytosolic DNA along with the induction of type-I IFNs through the activation of the cytosolic DNA sensing cGAS/STING pathway (4, 5). Consequently, dendritic cells (DCs) are recruited into the tumor microenvironment where they uptake tumor antigens, then they migrate to tumor draining lymph nodes (TDLNs) to prime or cross-prime naïve T cells (6, 7). Ensuing effector T cells are then recruited to the tumor site through chemokines secretion by tumor cells and other cell types in the tumor microenvironment (8, 9). Once T cells have infiltrated the tumor, they encounter tumor cells with radiation-induced expression or overexpression of several surface molecules and receptors, such as MHC-I molecules (10), TNF-R superfamily (11, 12) or ligands for the NKG2D receptor (13) leading to enhanced tumor cell killing by CD8^+^ T cells and NK cells. However, these immunostimulatory effects are counterbalanced by immunosuppression via several mechanisms, mainly upregulation of PD-L1 levels on tumor cells via INF-γ released by CD8^+^ T cells and of PD-1 levels on CD8^+^ tumor infiltrating lymphocytes (TILs), contributing to T-cell exhaustion (14, 15). Irradiation is also associated to direct depletion of circulating lymphocytes and lymphoid progenitors in primary and secondary lymphoid organs (16, 17) and to the enhancement of immune suppressive pathways within the tumor microenvironment such as those associated with induction of TGF-β and generation of adenosine from ATP (18). This dual effect can explain why irradiation alone is not systematically able to drive a strong antitumor immune response with a so-called “abscopal” effect, and underlies the rationale for combining radiotherapy to immunotherapy, not only to amplify the *in situ* vaccination effect but also to overrule immunosuppressive effects (19). Moreover, irradiation schedules need to be optimized to provoke *in situ* vaccination, and high doses per fraction (ideally 8 – 9 Gy) seem more likely associated to efficient antitumor immunity (10, 20, 21).

While much progress has been accomplished for the understanding of radiation-induced immune effects at the tumor level, little is known regarding the mechanisms involved at the TDLN level when the tumor is irradiated. In the context of stereotactic radiotherapy and high dose per fraction to induce abscopal effect, preclinical models have established the deleterious effect of TDLN irradiation combined to primary tumor irradiation, as TDLN irradiation was shown to attenuate the antitumor immune effects of radiotherapy, whether or not it is associated with immune checkpoint inhibitors (ICIs) (22–24). Several mechanisms following TDLN irradiation were described to explain such detrimental effect, mostly: modification of intratumoral T-cell chemoattractant chemokine signatures leading to decrease of immune cells infiltration within tumors, and especially antigen-specific CD8^+^ effector T cells; depletion of the “stem like” CD8^+^ T cell subset in both lymph nodes and tumor; decrease in epitope spreading and T-cell activation in distant lymph nodes (22–24).

Albeit crucial to the understanding of radiation-mediated *in situ* vaccination, the above cited preclinical data do not provide strong and direct clinical applications for the management of non-involved TDLNs during irradiation, for two main reasons. First, when radiotherapy aims at inducing a systemic effect through *in situ* vaccination, stereotactic irradiation with high dose per fraction is probably the most favorable approach, showing, nonetheless, contrasted but promising results in combination with ICI (25–27). In this approach, stereotactic radiotherapy is directed to metastatic lesions only, without elective lymph node (LN) irradiation, with low to no incidental dose to TDLNs due to the sharp dose fall-off gradient outside the target inherent to the stereotactic irradiation technique (28). Second, the question of non-involved TDLN sparing is rather addressed when large irradiation fields are employed, typically in the treatment of locally advanced disease. In these cases, chemotherapy is frequently combined to radiotherapy, and conventional fractionation is used (once-daily fractions of 2 – 3 Gy, five days a week, usually for 4 – 7 weeks), rather than ultra-hypofractionation (29). However, as compared to ultra-hypofractionated regimen (> 6 Gy per fraction), conventional fractionation has been shown in preclinical models to disturb the radiation immunostimulatory effects via the reduction of CD8^+^ T-cell infiltration, type-I IFN levels and MHC expression, the increase of PD-L1 expression and the increase of myeloid-derived suppressor cells recruitment into tumors through the VEGF pathway, which is inhibited in the context of hypofractionation (30, 31). Therefore, there is need to better understand the immune effects of chemoradiotherapy (CRT) at the TDLN level in the context of conventional fractionated irradiation for locally advanced tumors, to provide biological data in favor or not of the radiation dose sparing of non-involved, and therefore functional TDLNs.

To do so, we hypothesized that *non-involved* TDLNs among cN+ locally advanced non small cell lung cancer (NSCLC), downstream of involved TDLNs, share the same functional features as non-involved TDLNs from cN0 patients. We then proposed to focus on cN0 patients who received chemotherapy plus radiotherapy upon primary tumor only, in order to avoid incidental dose to non-involved TDLNs that inevitably occurs in the absence of specific dose constraints during mediastinal irradiation, which could disturb immune response in functional TDLNs. The objective of this retrospective study was to assess the immune and biological effects in non-involved TDLNs of node sparing CRT, by comparing TDLN gene expression signatures between patients with localized node-negative NSCLC treated either with upfront surgery (including LN dissection) (CRT^-^) or by neoadjuvant CRT (CRT^+^), whereby irradiation is directed at the primary tumor without incidental node irradiation, followed by surgery.

## Materials and methods

### Patient selection

Patients treated in two French cancer centers (Toulouse Cancer Center and Dijon Cancer Center) between January 2010 and December 2021 were identified through the electronical records database. The study was approved by the Institutional Review Board of our institution. Patients received a letter detailing the aim of the study and the use of data collection and could refuse inclusion at any time. Inclusion criteria were: histopathologically proved NSCLC; clinical N0,M0 (according to the eighth edition of the *Cancer Staging Manual* of the AJCC) with ^18^FDG-PET-based staging; treated by radical surgery (lobectomy) plus LN dissection with clinical T stage ≥ T2 and/or post-operative pathological T stage ≥ (y)pT2, and with pN0 on LN dissection; with (CRT^+^) or without (CRT^-^) neoadjuvant CRT of at least 44 Gy in 1.8 – 2 Gy per fraction, targeted on the primary tumor with no significant incidental irradiation of the TDLN station (*i.e.*, volume of the TDLN station receiving 20 Gy or more of less than 20% (V20 Gy < 20%)); Eastern Cooperative Oncology Group performance status < 2 at diagnosis; and with documented radiological follow-up for at least 6 months after surgery. Due to the rarity of neoadjuvant CRT in the management of NSCLC (32, 33), patients in the CRT^+^ group have been identified within the entire period whereas patients in the CRT^-^ group have been consecutively identified among those treated in 2017/2018. Patient data, including demographics, imaging, radiotherapy planning data and clinical outcome were retrospectively collected. The cut-off date for the analysis was March 2022.

### Definition of TDLN stations

According to the Mountain & Dresler regional LN mapping (34) as well as to post-mortem analysis of lung/bronchus LN draining system (35), and considering that subsegmental, segmental and lobar LNs (respectively stations XIV, XIII, and XII) are inconstantly present and/or identifiable in adults, the ipsilateral interlobar LNs (station XI) are considered as the first draining LNs of lung tumors. Therefore, we considered as TDLNs the ipsilateral interlobar LNs.

### Tissue samples and RNA isolation and sequencing

LN tissues were obtained from the pathological specimens of LN dissection. Rapidly after resection, LNs were placed in 10% neutral buffered formalin solution, fixed at room temperature and embedded in paraffin blocks (FFPE). One to three blocks were obtained for each patient. FFPE blocks were sliced into 4 µm unstained sections on glass slides for RNA extraction.

RNA was extracted using the RNeasy FFPE kit (Qiagen) following scraping of LN tissues with a razor blade from 15 glass slides per sample and deparaffinization using the Deparaffinization Solution (Qiagen). RNA concentration was quantified by spectrophotometry (NanoDrop™ One, Thermo Scientific) and fluorometry (Qubit™, Invitrogen), and RNA quality (DV200) was assessed using the Fragment Analyzer 5200 System (Agilent) and the DNF-472 HS RNA (15nt) kit (Agilent). Total RNA libraries were prepared using Illumina stranded Total RNA prep ligation with Ribo-Zero Plus kit (Illumina) starting with an input amount of 187-200 ng total RNA. The size and quality of the libraries were evaluated with the Fragment Analyzer 5200 System and the DNF-474 HS NGS Fragment kit (1-6000bp) (Agilent). The KAPA Library Quantification kit for Illumina platforms (KAPA Biosystems, Roche) was used to quantify the libraries. Samples were pooled in equimolarity. The double indexed libraries were sequenced on a NextSeq 550 instrument (Illumina) using 74 base-length read chemistry in paired-end mode.

For each sample, FASTQ raw files were trimmed with Trimgalore v0.4.5 then aligned to hg38 genome with STAR v2.7.5. MarkDuplicates (PicardTools v2.20.7) was used to remove PCR duplicates. Htseq-count (HTSeq v0.9.1) was used to quantify genes and counts were normalized in TPM (Transcript Per Million).

### Transcriptome data analysis

t-SNE analysis was performed using Rtsne v0.16 (R package). Differential gene expression (DGE) analysis was performed with DESeq2 (R package), and genes were considered differentially expressed if *P*-adjusted value ≤ 0.05 and log2 fold-change ≤ -1 or ≥ 1. Pathway enrichment was performed for significant genes with the Gene Set Enrichment Analysis (GSEA) and g-profiler software (https://biit.cs.ut.ee/gprofiler/gost) using the human collections of the Molecular Signature Database (MSigDB; https://www.gsea-msigdb.org/gsea/msigdb): hallmarks, curated gene sets (Biocarta, KEGG, PID and Reactome subsets), oncogenic, immunologic and cell-type signatures. For the sake of comparison, we performed additional analyses using gene signatures from pre-metastatic TDLNs (36–42).

Statistical analyses were performed using R v4.2.1. Plots were generated using ggplot2 v3.3.6 and ComplexHeatmap v2.13.1.

## Results

### Patient enrollment and sample selection

In total, 16 patients were included in the study and 25 TDLNs from station XI were analyzed: 6 patients in the CRT^+^ group (12 TDLNs) and 10 patients in the CRT^-^ group (13 TDLNs). The median age at diagnosis was 59 years in the CRT^+^ group and 66 years in CRT^-^ group. Adenocarcinoma was the main histology in both groups (83% in CRT^+^ and 90% in CRT^-^ group). Clinical stage was cT3 for all CRT^+^ patients; all patients in this group had superior sulcus tumor. In the CRT^-^ group, 2 patients (20%) had cT1, 6 patients (60%) had cT2 and 2 patients (20%) had cT3 tumor. In the CRT^-^ group, however, final pathological T stage was pT2 in 7 patients (70%) and pT3 in 3 patients (30%). In the CRT^+^ group, complete response and major pathological response occurred in respectively 1 patient (17%) and 3 patients (50%). CRT consisted of radiotherapy plus concomitant chemotherapy for all CRT^+^ patients, with a total of 3-4 cycles of platinum-based doublet; median dose of radiotherapy to the primary tumor was 47 Gy (44 – 50 Gy). Patients’ characteristics are summarized in **Table 1**.

**Table 1:** Clinical characteristics of the cohort.

	CRT^+^ group n=6 patients	CRT^-^ group n=10 patients
**Median age (y)**	59 (45 – 64)	66 (50 – 82)
**Male / Female**	6 / 0	6 / 4
**Median ECOG PS**	1 (0 – 1)	1 (0 – 1)
Histology		
- adenocarcinoma	5 (83%)	9 (90%)
- squamous cell carcinoma	1 (17%)	1 (10%)
Clinical T stage		
- T1	0	2 (20%)
- T2	0	6 (60%)
- T3	6 (100%)	2 (20%)
- T4	0	0
Patholgical T stage		
- (y)pT0	1 (17%)	0
- (y)pT1	2 (33%)	0
- (y)pT2	0	7 (70%)
- (y)pT3	3 (50%)	3 (30%)
Resection status		
R0	5 (83%)	10 (100%)
R1	1 (17%)	0
Pathological response (CRT^+^ group)		
Complete response	1 (17%)	na
Major pathological response	3 (50%)	na
Residual viable tumor > 10%	2 (33%)	na
**Median number of TDLN analyzed per patient**	2 (2 – 3)	1 (1 – 2)
**Median radiation total dose (Gy)**	47 (44 – 50)	na
**Median radiation dose per fraction (Gy)**	2	na
**Radiation technique**		na
Conformal 3D RT	1 (17%)	na
IMRT	5 (83%)	na
**Concomitant chemotherapy**	6 (100%)	na
**Median time interval between end of radiotherapy and surgery (days)**	93 (71 – 198)	na

na, not applicable.

### TDLNs from patients receiving neoadjuvant CRT and those treated with upfront surgery exhibited distinct molecular signatures

**Figure 1:**
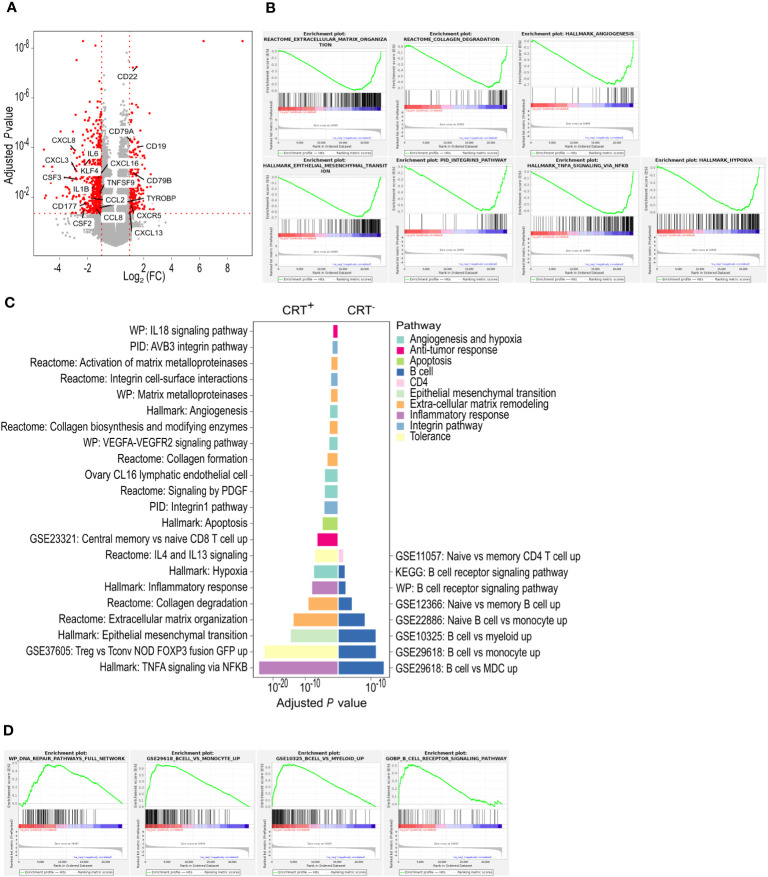
TDLNs from patients receiving neoadjuvant CRT and those treated with upfront surgery exhibit distinct molecular signatures. **(A)** Differentially expressed genes between CRT^+^ and CRT^-^ groups. In red, are represented genes with an adjusted *P*-value ≤ 0.05 and a log2 fold-change [Log2 (FC)] ≤ -1 or ≥ 1. All other genes are in grey. **(B)** CRT^+^ enriched pathways found with GSEA. **(C)** Enriched pathways found with g-profiler. Each pathway is colored depending on the broad biological processes in which it is involved. **(D)** CRT^-^ enriched pathways found with GSEA. n.a., not applicable.

Based on RNA sequencing (RNA-seq) data, we compared TDLNs from CRT^+^ and CRT^-^ patients. Analysis of differential gene expression uncovered a total of 1001 differentially expressed genes, of which 749 were more expressed in the CRT^+^ group and 252 were more expressed in the CRT^-^ group (**Figure 1A**). To gain insight into the major pathways that were differentially activated in each condition, we performed GSEA and g-profiler analyses, based on the 1001 differentially expressed genes.

In the CRT^+^ group, GSEA revealed that overall, 31 hallmark (C1), 192 canonical pathway (C2), 1232 ontology (C5), 118 oncogenic signature (C6), 1020 immune signature (C7) and 365 cell type signature (C8) gene sets were enriched, suggesting that tumor irradiation induced major changes in TDLNs (**Supplementary Table 1**). Among them, gene sets associated with epithelial mesenchymal transition, TNF-α signaling, hypoxia, angiogenesis, integrin pathway, collagen degradation and extra-cellular matrix remodeling were enriched (**Figure 1B**). G-profiler analysis revealed 549 enriched gene sets in the CRT^+^ group. Among them, gene sets associated with inflammatory response including TNF-α signaling, central memory CD8^+^ T cell (T_CM_) signature as well as immune tolerance (through regulatory CD4^+^ T-cell signature (Treg) and IL-4 signaling), all suggestive of an ongoing T-cell response, were enriched. The other enriched signatures included hypoxia, angiogenesis, apoptosis, epithelial mesenchymal transition, extra-cellular matrix remodeling and integrin pathway (**Figure 1C**).

In the CRT^-^ group, the number of enriched gene sets uncovered by GSEA was lower than that found in CRT^+^ TDLNs. Those enriched in CRT^-^ TDLNs included 2 hallmark (C1), 18 canonical pathway (C2), 31 ontology (C5), 135 immune signature (C7) and 39 cell type signature (C8) gene sets (**Supplementary Table 2**). Among enriched immune gene sets, those associated with B-cell responses stood out (**Figure 1D**). G-profiler analysis showed that the number of gene sets enriched in the CRT^-^ group, 79 in total, was lower than that found in the CRT^+^ group. In agreement with GSEA, gene sets associated with B-cell responses, including B-cell signature and B-cell receptor signaling, were identified by g-profiler analyses (**Figure 1C**).

Altogether, differential gene expression, GSEA and g-profiler analyses put forward a picture whereby neoadjuvant CRT led to changes in the immune molecular signatures of TDLNs. They showed the prominence of B-cell signatures in TDLNs from patients who received upfront surgery (CRT^-^ patients) whereas inflammatory, T-cell and immune tolerance signatures were higher in TDLNs from patients who received neoadjuvant CRT (CRT^+^ patients) (**Supplementary Figure 1**).

### Unsupervised dimensionality reduction identified a group of CRT^+^ patients with favorable clinical outcome and induction of a pre-metastatic TDLN signature

**Figure 2:**
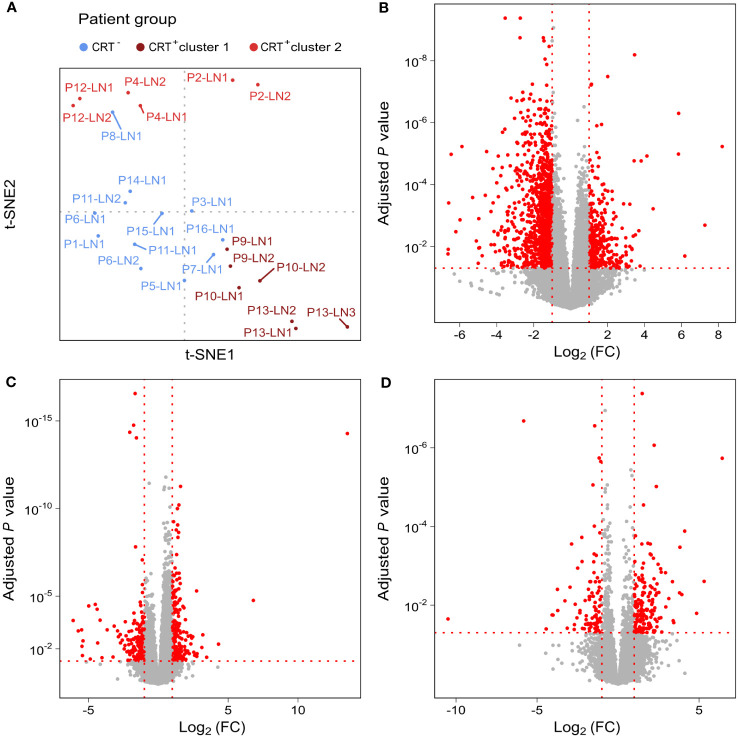
Unsupervised analysis shows two clusters in the CRT^+^ group. **(A)** t-SNE representation of TDLNs, colored depending on treatment received by patients. Differentially expressed genes between **(B)** CRT^+^ cluster 1 and all CRT^-^ samples, **(C)** CRT^+^ cluster 2 and all CRT^-^ samples and **(D)** CRT^+^ cluster 1 and CRT+ cluster 2. In red, are represented genes with an adjusted *P*-value ≤ 0.05 and a log2 fold-change [Log2 (FC)] ≤ -1 or ≥ 1. All other genes are in grey.

Unsupervised dimensionality reduction led to sample clustering according to the treatment group. CRT^+^ samples segregated into 2 clusters (clusters 1 and 2) and CRT^-^ samples formed a distinct cluster (**Figure 2A**). A total of 418 differentially expressed genes were identified when samples from CRT^+^ cluster 2 were compared to CRT^-^ samples, of which 210 were more expressed in cluster 2 and 208 in the CRT^-^ samples (**Figure 2B**). A higher number of genes, 1596, were found to be differentially expressed between CRT^+^ cluster 1 and CRT^-^ samples, of which the majority, 1210, were more expressed in CRT^+^ cluster 1 and 386 were more expressed in CRT^-^ samples (**Figure 2C**). In the 2 sets of genes found to be differentially expressed when each CRT^+^ cluster was compared to CRT^-^ samples, 174 were overlapping. Finally, a total of 256 differentially expressed genes were identified when samples from CRT^+^ cluster 1 and CRT^+^ cluster 2 were compared, of which 164 were more expressed in cluster 1 and 92 were more expressed in cluster 2 (**Figure 2D**).

**Figure 3:**
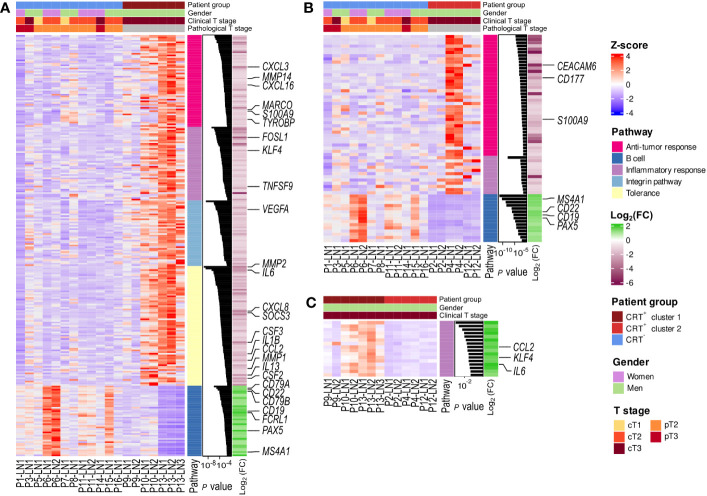
Enriched pathways in the two CRT^+^ clusters compared to CRT^-^ samples. Heatmap representation of the scaled expression (Z-score) of the differentially expressed genes, associated to the enriched pathways found with g-profiler **(A)** in the CRT^+^ cluster 1 and CRT^-^ comparison; **(B)** in the CRT^+^ cluster 2 and CRT^-^ comparison and **(C)** in the CRT^+^ cluster 1 and CRT^+^ cluster 1 comparison. Log2 (FC): log2 fold-change.

We then performed g-profiler analyses based on the 2 sets of differentially expressed genes. We identified 725 gene sets as being enriched in CRT^+^ cluster 1 when compared to CRT^-^ samples (**Figure 3A**). Those were involved in inflammatory response, antitumor response (including central memory (T_CM_) and effector memory (T_EM_) CD8^+^ T-cell signatures) as well as immune tolerance (including IL-4 and IL-10 signaling and Treg signatures), extra-cellular matrix remodeling, integrin pathway, angiogenesis and hypoxia, apoptosis, and epithelial mesenchymal transition. In addition, 83 gene sets enriched in CRT^+^ cluster 2 as compared to CRT^-^ samples were uncovered, mostly involved in antitumor response, inflammatory response, angiogenesis and hypoxia (**Figure 3B**). In both cases, gene sets associated with B-cell signatures were enriched in the CRT^-^ group. Finally, when CRT^+^ cluster 1 and CRT^+^ cluster 2 were compared, 121 gene sets were enriched in CRT^+^ cluster 1 (involved in inflammatory response, hypoxia, apoptosis, and epithelial mesenchymal transition but not in anti-tumor immune response), and 90 gene sets were enriched in CRT^+^ cluster 2 (involved in DNA damage repair) (**Figure 3C**).

Analysis of pathological response following neoadjuvant CRT revealed that among patients in CRT^+^ cluster 1, 3 out of 3 had pathological complete response (pCR) or major pathological response (MPR). In contrast, only 1 out of 3 patients in CRT^+^ cluster 2 had MPR whereas the 2 other patients had 30% and 60% residual viable tumor cells. Of note, mean time interval between end of radiotherapy and surgery was 127 days for patients in the CRT^+^ cluster 1 and 97 days for those in the CRT^+^ cluster 2, and median total radiation dose was 46 Gy for patients in CRT^+^ cluster 1 (46 – 48 Gy), and 46 Gy for patients in CRT^+^ cluster 2 (44 – 50 Gy). Characteristics of patients in cluster 1 and cluster 2 are summarized in **Supplementary Table 3**.

We then examined the expression, in CRT^+^ and CRT^-^ samples, of genes described as being involved in molecular changes within pre-metastatic TDLNs (36–42). Expression of TDLN-related genes involved in HEV (High Endothelial Venules) regulation and intra-node lymphocyte traffic as well as intra-node B cell traffic (*CXCR5*, *CXCL13*) was more prominent in CRT^-^ samples (**Supplementary Figure 2**). In the CRT^+^ group instead, particularly in samples within cluster 1, several genes involved in nodal lymphangiogenesis (*VEGFA*), fibroblastic reticular cells (FRC)-induced nodal remodeling (*PTX3*), tumor derived extracellular vesicles (tEV)-induced genes involved in tumor cell recruitment (*EPCAM*), matrix remodeling (*MMP2*, *PLAU*), angiogenesis (*THBS1*) and TDLN immune-suppression (*IL6*) (**Supplementary Figure 2**), all described as being upregulated in pre-metastatic TDLNs (36–42), were more expressed, suggesting that neoadjuvant tumor irradiation was involved in the remodeling of TDLNs in these patients.

## Discussion

Overall, in a population of patients with node-negative NSCLC, our data showed distinct transcriptomic patterns within non-involved TDLNs between patients who received or not neoadjuvant chemotherapy and radiotherapy of the primary tumor with conventional fractionation. Gene sets associated with several immunological as well as non-immunological pathways were enriched in patients having received neoadjuvant CRT (CRT^+^ group). This enrichment was pronounced in a subgroup of CRT^+^ patients experiencing pCR or MPR following neoadjuvant CRT.

Specifically in the CRT^+^ group, an enrichment was found in a gene set which is up regulated in a subset of early precursors of T_CM_ CD8^+^ T cells with enhanced self-renewal capacity and proliferative potential (43). This population is thought to represent the least differentiated memory T-cell subset and is commonly designated stem-cell like memory (T_SCM_) (44). Interestingly, Im et al. have suggested that the T-cell proliferative burst following blockade of PD-L1 was provided by progenitor exhausted T cells, which share the CD8^+^ T_SCM_ phenoype, found at high frequency in lymphoid organs, leading to more differentiated effectors (45). Additionally, the same team, in a subcutaneous B16F10 tumor model injected bilaterally in each flank, showed that radiotherapy directed toward one tumor could induce an abscopal response on the opposite flank (23). In this model, hypofractionated irradiation (1 fraction of 10 Gy) stimulated stem-like CD8^+^ T-cell proliferation, and the irradiation of tumor plus TDLNs reduced the abscopal effect, with decrease in this stem like population (23). Similarities between T_SCM_ and progenitor exhausted T cells have been hypothesized (46). In such case, our clinical data in NSCLC patients treated with conventional radiotherapy plus chemotherapy are in line with the preclinical data highlighting the role of radiotherapy in the proliferation of a stem like CD8^+^ T-cell population within TDLNs, with potential systemic anti-tumor effect.

On the other hand, potential tolerogenic pathways were found enriched in the CRT^+^ group. For example, IL-4 and IL-13 signaling pathways were enriched in the CRT^+^ group as well as IL-10 signaling pathway in CRT^+^ cluster 1. IL-4 and IL-13 are mainly involved in type 2 immunity and allergic diseases (47). In the context of malignancies, IL-4 and IL-13 have been shown to induce polarization towards protumoral tumor-associated macrophages (TAM) (48). However, IL-4 has also been involved in the maintenance of LN stromal organization (49). IL-10 is an immunosuppressive cytokine, which potentiates the differentiation of induced Tregs (50). Interestingly, a distinct Treg-related gene set, identified in *Foxp3* ^fgfp^ Treg from B6 mice (51), was also enriched in the CRT^+^ group. This mutant of *Foxp3* had a specific transcriptional profile with over-representation of IRF4 (Interferon Regulatory Factor 4)-dependent transcripts, leading to the preferential suppression of T-cell help to B cells. This could explain the underexpression of genes linked to B-cell signatures in the CRT^+^ group, in comparison to CRT^-^ patients. However, the global immunological effects of this pattern should be regarded cautiously. Indeed, it has been suggested that tumor growth was associated with accumulation of regulatory B cells (Breg) within TDLNs (52–54), which in turn could promote cancer cells recruitment via the production of anti-HSPA4 immunoglobulin and the activation of the CXCL12/CXCR4 pathway (55). Notably, *FCER2* which is a negative regulator of BCR signaling, is overexpressed in the CRT^-^ group (56).

Overall, our data show distinct non-involved TDLN immunological patterns between NSCLC patients who benefited or not from neoadjuvant CRT, with preferential strengthening in anti-tumoral immune effects following CRT. Tolerogenic patterns have been identified however after CRT as well, reflecting the dual effects of radiation on the immune response (19).

Non immunological gene sets were also assessed. Gene sets related to angiogenesis and hypoxia, epithelial mesenchymal transition and extra-cellular matrix remodeling were enriched in the CRT^+^ group. Surprisingly, these pathways have been described in the generation of a pre-metastatic niche, and specific genes described in such processes have been found in CRT^+^ samples. For example, VEGF-A is known to mediate lymphangiogenesis which in turn favors the afflux of tumor cells (37). *PTX3* was described as an actor of FRC-induced nodal remodeling which disrupts lymphocytes traffic and therefore immune response (39). *EPCAM*, *PLAU* and *THBS1* were shown to be induced within TDLNs secondary to the afflux of tEV from primary tumor, and are involved in tumor cell recruitment and extra-cellular matrix remodeling (42, 57–59). We assume that these pathways have been widely described in murine models, with no strict reproducibility in clinical samples.

Overall, our data indicate that CRT to the primary tumor provokes distinct microenvironment and immunological patterns within non-involved TDLNs. Hypothesizing that non-involved TDLNs from patients with (cN+) or without (cN0) nodes involvement share the same functional features, our study focusing on cN0 patients irradiated on the primary tumor only acts as a clinical model of node sparing-like CRT, with the guarantee of no incidental mediastinal node irradiation and a more reliable identification of TDLNs. Consequently, we suggest that our results could be extrapolable to locally advanced cN+ NSCLC patients who receive non-involved node sparing definitive conventional fractionation chemoradiation. Whether or not prophylactic nodal irradiation [commonly designated as elective node irradiation (ENI)] combined with primary tumor irradiation would disrupt immune response in the context of CRT ± ICIs for locally advanced tumors remains to be demonstrated. Two pre-clinical studies have shown that TDLN irradiation combined to the irradiation of the primary tumor with high dose fractionation (1 x 10 – 12 Gy) attenuates the anti-tumor immune effects of radiotherapy, whether or not it is associated with ICIs (22, 23). These effects were shown to be associated to decrease in immune cells within tumors, including antigen-specific CD8^+^ T cells, that could be explained by the direct depletion of specific stem-cell like CD8^+^ T cells within TDLNs. In a recent study using a preclinical model of head and neck squamous cell carcinoma (HNSCC), Darragh et al. showed that ENI to a dose of 8 Gy x 3 could disrupt local and systemic anti-tumor response following combined primary tumor radiation (3 x 8 Gy) and immunotherapy (anti-CD25) through: i) decrease in tumor-antigen specific T-cell priming in TDLNs and consequently decrease in circulating antigen-specific T cells and in infiltration into the tumor microenvironment, and ii) disruption of epitope spreading and T-cell priming at distant LNs. Furthermore, they validated these findings in canine patients treated with stereotactic tumor radiotherapy with or without ENI. They found that TDLNs treated with ENI showed decrease in transcription of genes associated with antigen presentation, with effector T cells and with T-cell homing. Finally, they assessed human patients with HNSCC from a phase I/Ib clinical trial of neoadjuvant stereotactic hypofractionated radiotherapy to the tumor only (2 or 3 fractions of 6 Gy) with durvalumab followed by neck dissection at 3 to 6 weeks. The non–irradiated TDLNs from patients enrolled in the trial were compared to normal nodes from non-treated patients. By avoiding ENI, non-irradiated TDLNs showed activated T cells defined by increase in IFN-γ expression (24).

Albeit crucial, these preclinical and early-clinical data cannot be extrapolated in the context of CRT ± ICIs for locally advanced disease, and especially NSCLC, because in the latter case: i) conventional fractionation is usually used rather than extreme hypofractionation to both primary tumor and TDLNs when ENI is performed, ii) large fields are treated rather than small volumes, and iii) chemotherapy is combined to radiation. All of these could modify radiation-induced immune effects (60). In a pragmatic approach, changes within non-involved TDLNs have been addressed herein following radiotherapy using conventional fractionation and chemotherapy. Moreover, all the patients of our series were staged with 18FDG PET, which is associated with negative predictive value of nodal involvement of approximately 90% in localized disease (61). In addition, pathological examination confirmed the absence of involvement in both groups, albeit following neoadjuvant CRT in the CRT^+^ group. In comparison, the negative metastatic status of TDLNs in mice from the three studies (22–24) has not been confirmed, and the presence of metastatic tumor cells can intrinsically modify TDLN molecular patterns.

Our study has several limits that need to be highlighted. First, the retrospective nature of the study is an intrinsic limit. Second, the size of the cohort was limited although a total of 4 cancer centers have been involved in the screening process. Indeed, neoadjuvant CRT on the primary tumor only in NSCLC patients with cN0 is an unusual approach, that however could guarantee the absence of effects from nodal cancer cells and from direct radiation of TDLNs. Moreover, we cannot exclude that CRT^+^ patients had slightly more aggressive tumors, with more unfavorable microenvironment patterns. Nevertheless, all CRT^+^ patients had cT3 tumors, whereas all CRT^-^ patients had pT2 or pT3 tumors, and it seems unlikely that this slight imbalance is enough to explain the observed difference in TDLN signatures. Finally, the identification of TDLNs cannot be optimal as SLNs were not validated in NSCLC. Rather, we identified ipsilateral nodes (station XI)as reasonable alternative to SLNs in the series (34, 35). Naturally, the clinical benefit of this node sparing-like CRT on pathological tumor response in cluster 1 should be considered cautiously due to the low sample size. Overall, due to the sample size of our cohort, our data are exploratory at this stage, and would need to be confirmed in a larger cohort.

Notwithstanding these limitations, to our knowledge, our data represent the first attempt to decipher molecular changes in non-involved TDLNs following node sparing-like CRT in clinical practice in the context of NSCLC. Although exploratory, our data, in line with a previous study (24), provide new insights and potential rationale to reduce ENI practice, and even to apply stringent radiation dose constraints on non-involved TDLNs, in the context of conventional fractionated CRT + ICIs for locally advanced disease. Our results could explain the contrasting clinical outcomes of clinical trials assessing CRT for locally advanced tumors in association to ICIs. While the PACIFIC trial in NSCLC is the only phase III trial to have shown a benefit of the adjunction of ICIs with durvalumab to CRT for locally advanced disease, the JAVELIN trial in locally advanced HNSCC assessing the adjunction of avelumab to CRT failed to improve outcome (62), as well as the KEYNOTE-412 with pembrolizumab in HNSCC (NCT03040999) and the CALLA trial with durvalumab in cervical cancer (NCT03830866), according to recent press releases (63, 64). One of the key differences between the PACIFIC trial and the other negative trials is the absence of ENI in PACIFIC while it was used in the other trials. In line with this concept, we have shown that irradiation of at least one non-involved TDLN station was associated, in multivariable analysis, to poor clinical outcome following concurrent chemoradiation and consolidative immunotherapy of locally-advanced NSCLC (65). Overall, all these data provide a rational for avoiding ENI in the context of conventional fractionated CRT + ICIs for locally advanced disease, and even more to perform radiation dose sparing to non involved TDLNs, which can be considered as immune organs at risk for radiotherapy planning.

## Data availability statement

The datasets presented in this study can be found in online repositories. The names of the repository/repositories and accession number(s) can be found below: https://www.ncbi.nlm.nih.gov/geo/, GSE239514.

## Ethics statement

The studies involving humans were approved by IRB local committee, Institut Universitaire du Cancer de toulouse-Oncopole. The studies were conducted in accordance with the local legislation and institutional requirements. The ethics committee/institutional review board waived the requirement of written informed consent for participation from the participants or the participants’ legal guardians/next of kin because All the requirements to waive the informed consent were gathered: the research involved no more than minimal risk to subjects; the research could not be carried out practicably without the waiver or alteration; the waiver or alteration have not adversely affected the rights and welfare of the subjects; and the subjects have been provided with additional information about their participation (patients received a letter detailing the aim of the study and the use of data collection and could refuse inclusion at any time).

## Author contributions

JK: Conceptualization, Data curation, Formal analysis, Investigation, Methodology, Writing – original draft, Writing – review & editing. NT: Data curation, Formal analysis, Methodology, Software, Writing – review & editing. C-MS: Software, Visualization, Writing – review & editing. EN: Data curation, Project administration, Writing – review & editing. CM: Writing – review & editing. LB: Writing – review & editing. SP: Project administration, Writing – review & editing. CC: Project administration, Writing – review & editing. LV: Project administration, Writing – review & editing. CV: Data curation, Investigation, Writing – review & editing. ES: Data curation, Investigation, Writing – review & editing. NS-L: Data curation, Investigation, Writing – review & editing. EM: Data curation, Writing – review & editing. P-BP: Data curation, Writing – review & editing. AM: Data curation, Writing – review & editing. JM: Writing – review & editing. EC-J: Validation, Writing – review & editing. FL-V: Data curation, Formal analysis, Investigation, Writing – review & editing. MA: Investigation, Methodology, Supervision, Validation, Writing – review & editing.

## Supplementary material

Supplementary Figure 1 G-profiler enrichment analysis from genes differentially expressed in TDLNs from patients receiving neoadjuvant CRT. Heatmap representation of the scaled expression (Z-score) of genes differentially expressed in CRT^+^ and CRT^-^ samples and associated to the enriched pathways found with g-profiler.

Supplementary Figure 2 Expression, in CRT^+^ and CRT^-^ samples, of genes associated to pre-metastatic TDLNs. Heatmap representation of the scaled expression (Z-score) of genes associated to pre-metastatic TDLNs (36–42) in CRT^+^ and CRT^-^ samples and with a P-value ≤ 0.05. Genes significantly differentially expressed indicated by an asterisk. Log2 (FC): log2 fold-change.

Supplementary Table 1 Enriched gene sets in CRT+ group (GSEA analysis). ES, Enrichment Score; NES, Normalized Enrichment Score; NOM p value, Nominal *P*-value; FDR q value, adjusted *P*-value according to False Discovery Rate method (used in our analysis); FWER p value, adjusted *P*-value according to the Family Wise Error Rate.

Supplementary Table 2 Enriched gene sets in CRT- group (GSEA analysis). ES, Enrichment Score; NES, Normalized Enrichment Score; NOM p value, Nominal *P*-value; FDR q value, adjusted *P*-value according to False Discovery Rate method (used in our analysis); FWER p value, adjusted *P*-value according to the Family Wise Error Rate.

Supplementary Table 3 Patients’ characteristics in cluster 1 and cluster 2.

## References

[1] MOLE RH. Whole body irradiation—Radiobiology or medicine? Br J Radiol (2014) 26:234–41. doi: 10.1259/0007-1285-26-305-234 13042090

[2] Formenti SC Demaria S. Radiation therapy to convert the tumor into an *in situ* vaccine. Int J Radiat Oncol Biol Phys (2012) 84:879–80. doi: 10.1016/J.IJROBP.2012.06.020 PMC3811126 23078897

[3] Demaria S Ng B Devitt ML Babb JS Kawashima N Liebes L. Ionizing radiation inhibition of distant untreated tumors (abscopal effect) is immune mediated. Int J Radiat Oncol Biol Phys (2004) 58:862–70. doi: 10.1016/J.IJROBP.2003.09.012 14967443

[4] Weichselbaum RR Liang H Deng L Fu YX. Radiotherapy and immunotherapy: a beneficial liaison? Nat Rev Clin Oncol (2017) 14:365–79. doi: 10.1038/NRCLINONC.2016.211 28094262

[5] Deng L Liang H Xu M Yang X Burnette B Arina A. STING-dependent cytosolic DNA sensing promotes radiation-induced type I interferon-dependent antitumor immunity in immunogenic tumors. Immunity (2014) 41:843–52. doi: 10.1016/j.immuni.2014.10.019 PMC5155593 25517616

[6] Apetoh L Ghiringhelli F Tesniere A Obeid M Ortiz C Criollo A. Toll-like receptor 4-dependent contribution of the immune system to anticancer chemotherapy and radiotherapy. Nat Med (2007) 13:1050–9. doi: 10.1038/nm1622 17704786

[7] Gupta A Probst HC Vuong V Landshammer A Muth S Yagita H. Radiotherapy promotes tumor-specific effector CD8+ T cells *via* dendritic cell activation. J Immunol (2012) 189:558–66. doi: 10.4049/jimmunol.1200563 22685313

[8] Matsumura S Wang B Kawashima N Braunstein S Badura M Cameron TO. Radiation-induced CXCL16 release by breast cancer cells attracts effector T cells. J Immunol (2008) 181:3099–107. doi: 10.4049/JIMMUNOL.181.5.3099 PMC2587101 18713980

[9] Lim JYH Gerber SA Murphy SP Lord EM. Type I interferons induced by radiation therapy mediate recruitment and effector function of CD8(+) T cells. Cancer Immunol Immunother (2014) 63:259–71. doi: 10.1007/s00262-013-1506-7 PMC3944132 24357146

[10] Reits EA Hodge JW Herberts CA Groothuis TA Chakraborty M Wansley EK. Radiation modulates the peptide repertoire, enhances MHC class I expression, and induces successful antitumor immunotherapy. J Exp Med (2006) 203:1259–71. doi: 10.1084/jem.20052494 PMC3212727 16636135

[11] Chakraborty M Abrams SI Coleman CN Camphausen K Schlom J Hodge JW. External beam radiation of tumors alters phenotype of tumor cells to render them susceptible to vaccine-mediated T-cell killing. Cancer Res (2004) 64:4328–37. doi: 10.1158/0008-5472.CAN-04-0073 15205348

[12] Chakraborty M Abrams SI Camphausen K Liu K Scott T Coleman CN. Irradiation of tumor cells up-regulates fas and enhances CTL lytic activity and CTL adoptive immunotherapy. J Immunol (2003) 170:6338–47. doi: 10.4049/JIMMUNOL.170.12.6338 12794167

[13] Gasser S Orsulic S Brown EJ Raulet DH. The DNA damage pathway regulates innate immune system ligands of the NKG2D receptor. Nat (2005) 436:1186–90. doi: 10.1038/nature03884 PMC1352168 15995699

[14] Dovedi SJ Adlard AL Lipowska-Bhalla G McKenna C Jones S Cheadle EJ. Acquired resistance to fractionated radiotherapy can be overcome by concurrent PD-L1 blockade. Cancer Res (2014) 74:5458–68. doi: 10.1158/0008-5472.CAN-14-1258 25274032

[15] Deng L Liang H Burnette B Beckett M Darga T Weichselbaum RR. Irradiation and anti-PD-L1 treatment synergistically promote antitumor immunity in mice. J Clin Invest (2014) 124:687–95. doi: 10.1172/JCI67313 PMC3904601 24382348

[16] Venkatesulu BP Mallick S Lin SH Krishnan S. A systematic review of the influence of radiation-induced lymphopenia on survival outcomes in solid tumors. Crit Rev Oncol Hematol (2018) 123:42–51. doi: 10.1016/j.critrevonc.2018.01.003 29482778

[17] Cesaire M Le Mauff B Rambeau A Toutirais O Thariat J. Mécanismes de la lymphopénie radio-induite et implications thérapeutiques. Bull Cancer (2020) 107:813–22. doi: 10.1016/J.BULCAN.2020.04.009 32451070

[18] Wennerberg E Lhuillier C Vanpouille-Box C Pilones KA García-Martínez E Rudqvist N-P. Barriers to radiation-induced *in situ* tumor vaccination. Front Immunol (2017) 8:229. doi: 10.3389/fimmu.2017.00229 28348554 PMC5346586

[19] Walle T Martinez Monge R Cerwenka A Ajona D Melero I Lecanda F. Radiation effects on antitumor immune responses: current perspectives and challenges. Ther Adv Med Oncol (2018) 10:1758834017742575. doi: 10.1177/1758834017742575 29383033 PMC5784573

[20] Golden EB Frances D Pellicciotta I Demaria S Barcellos-Hoff MH Formenti SC. Radiation fosters dose-dependent and chemotherapy-induced immunogenic cell death. Oncoimmunology (2014) 3. doi: 10.4161/ONCI.28518/SUPPL_FILE/KONI_A_10928518_SM0001.ZIP PMC4106151 25071979

[21] Dewan MZ Galloway AE Kawashima N Dewyngaert JK Babb JS Formenti SC. Fractionated but not single-dose radiotherapy induces an immune-mediated abscopal effect when combined with anti-CTLA-4 antibody. Clin Cancer Res (2009) 15:5379–88. doi: 10.1158/1078-0432.CCR-09-0265 PMC2746048 19706802

[22] Marciscano AE Ghasemzadeh A Nirschl TR Theodros D Kochel CM Francica BJ. Elective nodal irradiation attenuates the combinatorial efficacy of stereotactic radiation therapy and immunotherapy. Clin Cancer Res (2018) 24:5058–71. doi: 10.1158/1078-0432.CCR-17-3427 PMC6532976 29898992

[23] Buchwald ZS Nasti TH Lee J Eberhardt CS Wieland A Im SJ. Tumor-draining lymph node is important for a robust abscopal effect stimulated by radiotherapy. J Immunother Cancer (2020) 8. doi: 10.1136/jitc-2020-000867 PMC7542667 33028691

[24] Darragh LB Gadwa J Pham TT Van Court B Neupert B Olimpo NA. Elective nodal irradiation mitigates local and systemic immunity generated by combination radiation and immunotherapy in head and neck tumors. Nat Commun (2022) 13:7015. doi: 10.1038/S41467-022-34676-W 36385142 PMC9668826

[25] Theelen WSME Peulen HMU Lalezari F van der Noort V de Vries JF Aerts JGJV. Effect of pembrolizumab after stereotactic body radiotherapy vs pembrolizumab alone on tumor response in patients with advanced non-small cell lung cancer: results of the PEMBRO-RT phase 2 randomized clinical trial. JAMA Oncol (2019) 5. doi: 10.1001/jamaoncol.2019.1478 PMC6624814 31294749

[26] Theelen WSME Chen D Verma V Hobbs BP Peulen HMU Aerts JGJV. Pembrolizumab with or without radiotherapy for metastatic non-small-cell lung cancer: a pooled analysis of two randomised trials. Lancet Respir Med (2020). doi: 10.1016/S2213-2600(20)30391-X 33096027

[27] Welsh J Menon H Chen D Verma V Tang C Altan M. Pembrolizumab with or without radiation therapy for metastatic non-small cell lung cancer: a randomized phase I/II trial. J Immunother Cancer (2020) 8. doi: 10.1136/jitc-2020-001001 PMC7555111 33051340

[28] Martin A Gaya A. Stereotactic body radiotherapy: a review. Clin Oncol (R Coll Radiol) (2010) 22:157–72. doi: 10.1016/J.CLON.2009.12.003 20092981

[29] Joiner M van der Kogel A. Basic clinical radiobiology. Hodder Arnold (2009). doi: 10.1201/b15450

[30] Morisada M Clavijo PE Moore E Sun L Chamberlin M Van Waes C. PD-1 blockade reverses adaptive immune resistance induced by high-dose hypofractionated but not low-dose daily fractionated radiation. Oncoimmunology (2018) 7:e1395996. doi: 10.1080/2162402X.2017.1395996 29399393 PMC5790397

[31] Lan J Li R Yin L-M Deng L Gui J Chen B-Q. Targeting myeloid-derived suppressor cells and programmed death ligand 1 confers therapeutic advantage of ablative hypofractionated radiation therapy compared with conventional fractionated radiation therapy. Int J Radiat Oncol Biol Phys (2018) 101:74–87. doi: 10.1016/j.ijrobp.2018.01.071 29619980

[32] Pless M Stupp R Ris H-B Stahel RA Weder W Thierstein S. Induction chemoradiation in stage IIIA/N2 non-small-cell lung cancer: a phase 3 randomised trial. Lancet (London England) (2015) 386:1049–56. doi: 10.1016/S0140-6736(15)60294-X 26275735

[33] Thomas M Rübe C Hoffknecht P Macha HN Freitag L Linder A. Effect of preoperative chemoradiation in addition to preoperative chemotherapy: a randomised trial in stage III non-small-cell lung cancer. Lancet Oncol (2008) 9:636–48. doi: 10.1016/S1470-2045(08)70156-6 18583190

[34] Mountain CF Dresler CM. Regional lymph node classification for lung cancer staging. Chest (1997) 111:1718–23. doi: 10.1378/chest.111.6.1718 9187199

[35] Riquet M. Cancer bronchique : le drainage lymphatique. Cancer/Radiotherapie (2007) 11:4–10. doi: 10.1016/J.CANRAD.2006.07.005 16928459

[36] du Bois H Heim TA Lund AW. Tumor-draining lymph nodes: At the crossroads of metastasis and immunity. Sci Immunol (2021) 6. doi: 10.1126/SCIIMMUNOL.ABG3551 PMC8628268 34516744

[37] Hirakawa S Kodama S Kunstfeld R Kajiya K Brown LF Detmar M. VEGF-A induces tumor and sentinel lymph node lymphangiogenesis and promotes lymphatic metastasis. J Exp Med (2005) 201:1089–99. doi: 10.1084/JEM.20041896 PMC2213132 15809353

[38] Bekkhus T Martikainen T Olofsson A Boger MF Bacovia DV Wärnberg F. Remodeling of the lymph node high endothelial venules reflects tumor invasiveness in breast cancer and is associated with dysregulation of perivascular stromal cells. Cancers (Basel) (2021) 13:1–17. doi: 10.3390/CANCERS13020211 PMC7827313 33430113

[39] Riedel A Shorthouse D Haas L Hall BA Shields J. Tumor-induced stromal reprogramming drives lymph node transformation. Nat Immunol (2016) 17:1118–27. doi: 10.1038/NI.3492 PMC4994871 27400148

[40] Kim R Emi M Tanabe K Arihiro K. Tumor-driven evolution of immunosuppressive networks during Malignant progression. Cancer Res (2006) 66:5527–36. doi: 10.1158/0008-5472.CAN-05-4128 16740684

[41] Nogués L Benito-Martin A Hergueta-Redondo M Peinado H. The influence of tumour-derived extracellular vesicles on local and distal metastatic dissemination. Mol Aspects Med (2018) 60:15–26. doi: 10.1016/J.MAM.2017.11.012 29196097 PMC5856602

[42] Hood JL San Roman S Wickline SA. Exosomes released by melanoma cells prepare sentinel lymph nodes for tumor metastasis. Cancer Res (2011) 71:3792–801. doi: 10.1158/0008-5472.CAN-10-4455 21478294

[43] Gattinoni L Lugli E Ji Y Pos Z Paulos CM Quigley MF. A human memory T cell subset with stem cell-like properties. Nat Med (2011) 17:1290–7. doi: 10.1038/NM.2446 PMC3192229 21926977

[44] Gattinoni L Speiser DE Lichterfeld M Bonini C. T memory stem cells in health and disease. Nat Med (2017) 23:18–27. doi: 10.1038/NM.4241 28060797 PMC6354775

[45] Im SJ Hashimoto M Gerner MY Lee J Kissick HT Burger MC. Defining CD8+ T cells that provide the proliferative burst after PD-1 therapy. Nature (2016) 537:417–21. doi: 10.1038/NATURE19330 PMC5297183 27501248

[46] Blank CU Haining WN Held W Hogan PG Kallies A Lugli E. Defining “T cell exhaustion.” Nat Rev Immunol (2019) 19:665–74. doi: 10.1038/S41577-019-0221-9 PMC7286441 31570879

[47] Bao K Reinhardt RL. The differential expression of IL-4 and IL-13 and its impact on type-2 immunity. Cytokine (2015) 75:25–37. doi: 10.1016/J.CYTO.2015.05.008 26073683 PMC5118948

[48] Zhou J Tang Z Gao S Li C Feng Y Zhou X. Tumor-associated macrophages: recent insights and therapies. Front Oncol (2020) 10:188. doi: 10.3389/FONC.2020.00188 32161718 PMC7052362

[49] Cortes-Selva D Ready A Gibbs L Rajwa B Fairfax KC. IL-4 promotes stromal cell expansion and is critical for development of a type-2, but not a type 1 immune response. Eur J Immunol (2019) 49:428. doi: 10.1002/EJI.201847789 30575951 PMC6953475

[50] Hsu P Santner-Nanan B Hu M Skarratt K Lee CH Stormon M. IL-10 potentiates differentiation of human induced regulatory T cells *via* STAT3 and foxo1. J Immunol (2015) 195:3665–74. doi: 10.4049/JIMMUNOL.1402898/-/DCSUPPLEMENTAL 26363058

[51] Darce J Rudra D Li L Nishio J Cipolletta D Rudensky AY. An N-terminal mutation of the Foxp3 transcription factor alleviates arthritis but exacerbates diabetes. Immunity (2012) 36:731–41. doi: 10.1016/J.IMMUNI.2012.04.007 PMC3386606 22579475

[52] Ruddell A Harrell MI Furuya M Kirschbaum SB Iritani BM. B lymphocytes promote lymphogenous metastasis of lymphoma and melanoma. Neoplasia (2011) 13:748–57. doi: 10.1593/NEO.11756 PMC3156665 21847366

[53] Harrell MI Iritani BM Ruddell A. Tumor-induced sentinel lymph node lymphangiogenesis and increased lymph flow precede melanoma metastasis. Am J Pathol (2007) 170:774–86. doi: 10.2353/AJPATH.2007.060761 PMC1851877 17255343

[54] Ganti SN Albershardt TC Iritani BM Ruddell A. Regulatory B cells preferentially accumulate in tumor-draining lymph nodes and promote tumor growth. Sci Rep (2015) 5:1–8. doi: 10.1038/srep12255 PMC4507466 26193241

[55] Gu Y Liu Y Fu L Zhai L Zhu J Han Y. Tumor-educated B cells selectively promote breast cancer lymph node metastasis by HSPA4-targeting IgG. Nat Med (2019) 25:312–22. doi: 10.1038/s41591-018-0309-y 30643287

[56] Liu C Richard K Wiggins M Zhu X Conrad DH Song W. CD23 can negatively regulate B-cell receptor signaling. Sci Rep (2016) 6. doi: 10.1038/SREP25629 PMC4867583 27181049

[57] Liu D Li C Trojanowicz B Li X Shi D Zhan C. CD97 promotion of gastric carcinoma lymphatic metastasis is exosome dependent. Gastric Cancer (2016) 19:754–66. doi: 10.1007/S10120-015-0523-Y PMC4906076 26233326

[58] Jung T Castellana D Klingbeil P Hernández IC Vitacolonna M Orlicky DJ. CD44v6 dependence of premetastatic niche preparation by exosomes. Neoplasia (2009) 11:1093–105. doi: 10.1593/NEO.09822 PMC2745675 19794968

[59] Rana S Malinowska K Zöller M. Exosomal tumor microRNA modulates premetastatic organ cells. Neoplasia (2013) 15:281–95. doi: 10.1593/NEO.122010 PMC3593151 23479506

[60] Khalifa J Mazieres J Gomez-Roca C Ayyoub M Moyal ECJ. Radiotherapy in the era of immunotherapy with a focus on non-small-cell lung cancer: time to revisit ancient dogmas? Front Oncol (2021) 11:662236. doi: 10.3389/FONC.2021.662236 33968769 PMC8097090

[61] Şanli M Isik AF Zincirkeser S Elbek O Mete A Tuncozgur B. Reliability of positron emission tomography–computed tomography in identification of mediastinal lymph node status in patients with non–small cell lung cancer. J Thorac Cardiovasc Surg (2009) 138:1200–5. doi: 10.1016/J.JTCVS.2009.03.035 19660381

[62] Lee NY Ferris RL Psyrri A Haddad RI Tahara M Bourhis J. Avelumab plus standard-of-care chemoradiotherapy versus chemoradiotherapy alone in patients with locally advanced squamous cell carcinoma of the head and neck: a randomised, double-blind, placebo-controlled, multicentre, phase 3 trial. Lancet Oncol (2021) 22:450–62. doi: 10.1016/S1470-2045(20)30737-3 33794205

[63] Merck provides update on phase 3 KEYNOTE-412 trial in unresected locally advanced head and neck squamous cell carcinoma - merck.com . Available at: https://www.merck.com/news/merck-provides-update-on-phase-3-keynote-412-trial-in-unresected-locally-advanced-head-and-neck-squamous-cell-carcinoma (Accessed July 31, 2022).

[64] Update on CALLA Phase III trial of concurrent use of Imfinzi and chemoradiotherapy in locally advanced cervical cancer . Available at: https://www.astrazeneca.com/media-centre/press-releases/2022/update-on-calla-phase-iii-trial-for-imfinzi.html (Accessed July 31, 2022).

[65] Pasquier C Chaltiel L Massabeau C Rabeau A Lebas L Lusque A. Impact of radiation on host immune system in patients treated with chemoradiotherapy and durvalumab consolidation for unresectable locally advanced non-small cell lung cancer. Front Oncol (2023) 13:1186479. doi: 10.3389/FONC.2023.1186479 37397359 PMC10313116

