# Supplementary figures and images for: Immune changes in hilar tumor draining lymph nodes following node sparing neoadjuvant chemoradiotherapy of localized cN0 non-small cell lung cancer
Source: Front Oncol. 2023 Nov 22;13:1269166. doi: 10.3389/fonc.2023.1269166 (PMC10699862; doi:10.3389/fonc.2023.1269166)

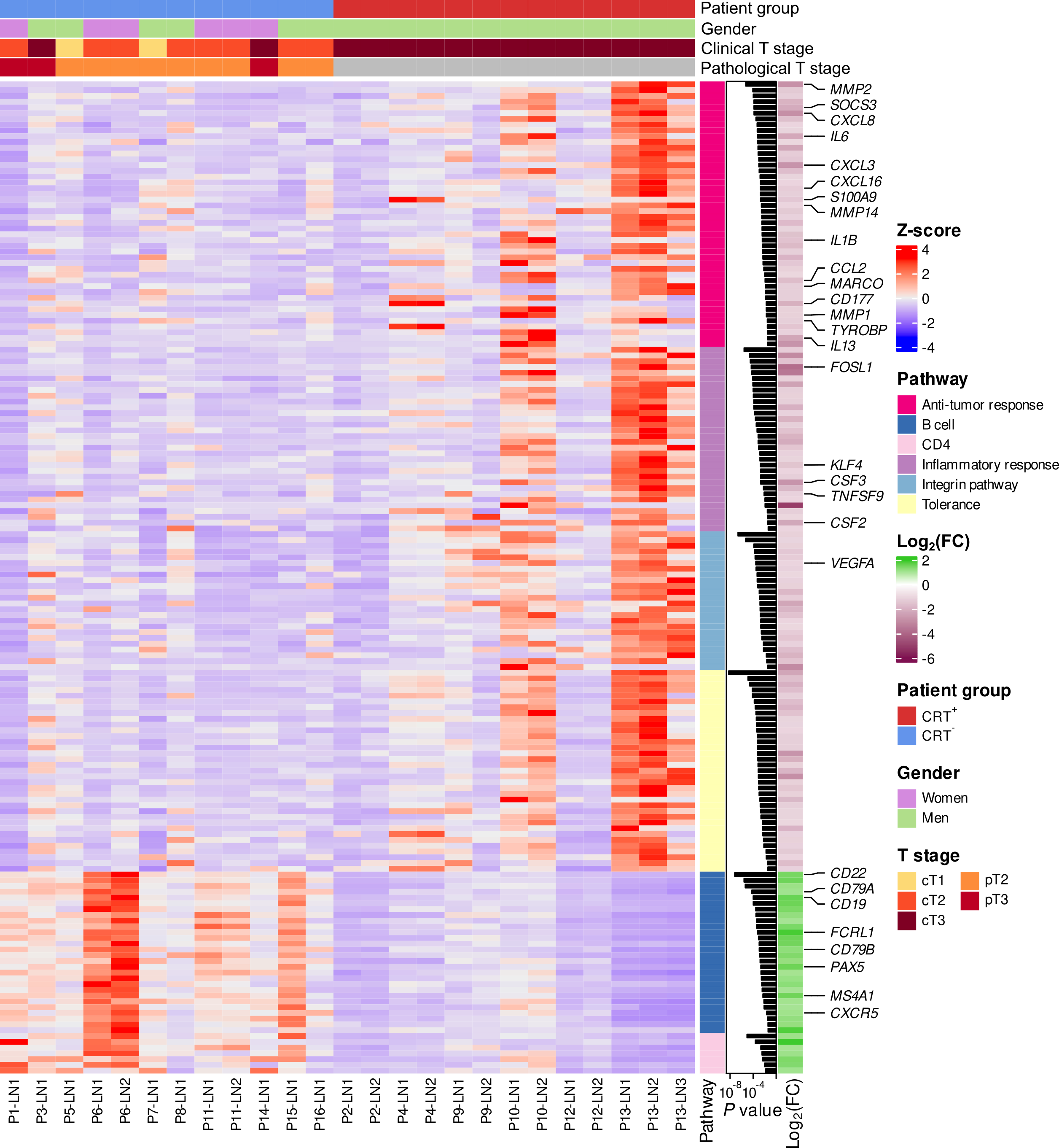

Supplement: Supplementary Figure 1 — G-profiler enrichment analysis from genes differentially expressed in TDLNs from patients receiving neoadjuvant CRT. Heatmap representation of the scaled expression (Z-score) of genes differentially expressed in CRT+ and CRT- samples and associated to the enriched pathways found with g-profiler. [file Image_1.tiff]

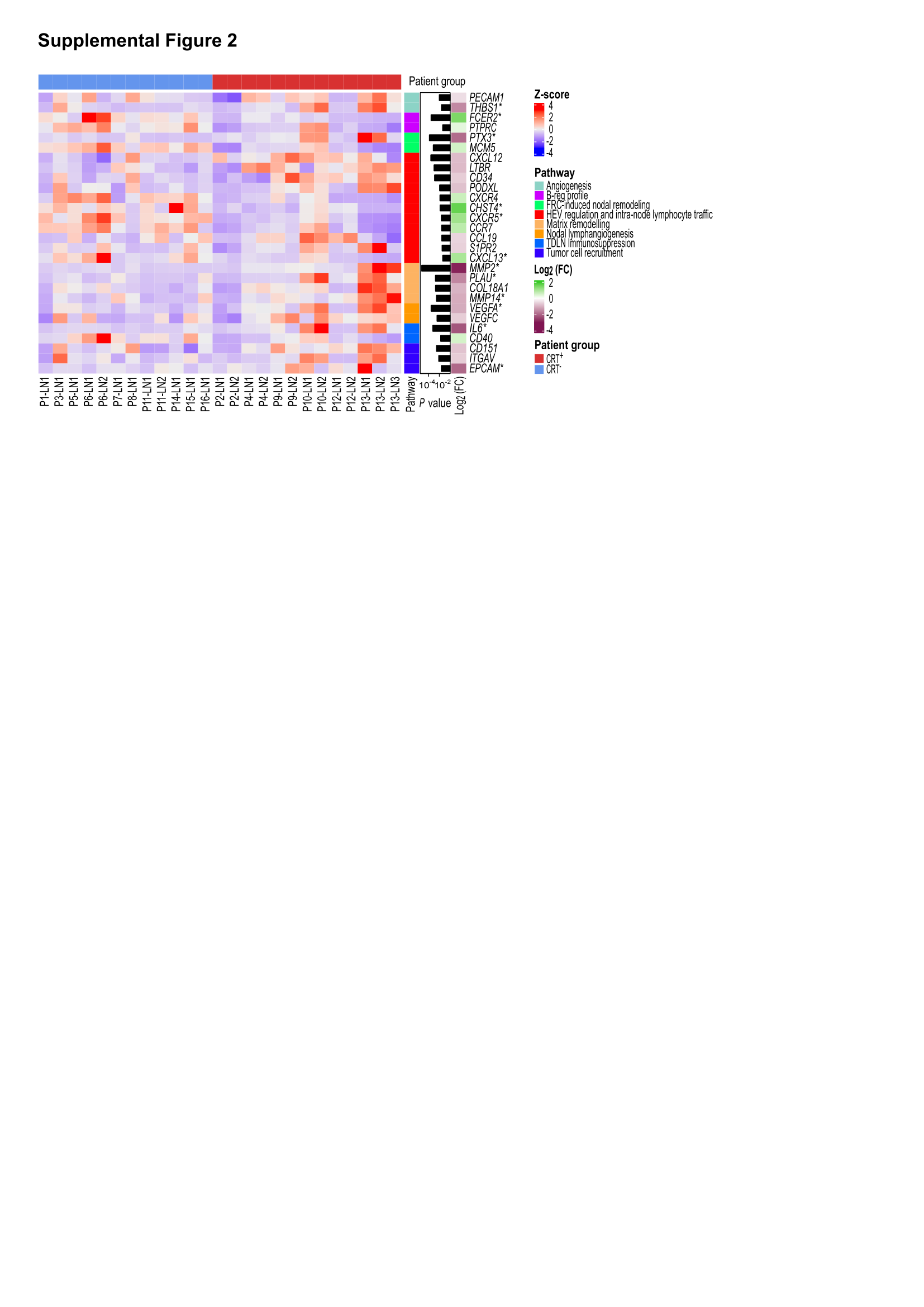

Supplement: Supplementary Figure 2 — Expression, in CRT+ and CRT- samples, of genes associated to pre-metastatic TDLNs. Heatmap representation of the scaled expression (Z-score) of genes associated to pre-metastatic TDLNs (36–42) in CRT+ and CRT- samples and with a P-value ≤ 0.05. Genes significantly differentially expressed indicated by an asterisk. Log2 (FC): log2 fold-change. [file Image_2.tiff]
