# Supplementary material for: Immune changes in hilar tumor draining lymph nodes following node sparing neoadjuvant chemoradiotherapy of localized cN0 non-small cell lung cancer
Source: Front Oncol. 2023 Nov 22;13:1269166. doi: 10.3389/fonc.2023.1269166 (PMC10699862; doi:10.3389/fonc.2023.1269166)
Supplement: Supplementary Table 3 — Patients’ characteristics in cluster 1 and cluster 2. [file Table_3.docx]

Supplementary Table 3

Patients’ characteristics in cluster 1 and cluster 2

|  | CRT^+^ group  cluster 1 (3 pts) | CRT^+^ group  cluster 2 (3 pts) |
| --- | --- | --- |
| Median age (y) | 58 | 60 |
| Median ECOG PS | 1 (0 – 1) | 1 ( 1 – 1) |
| Histology |  |  |
| - adenocarcinoma | 3 | 2 |
| - squamous cell carcinoma | 0 | 1 |
| Clinical T stage |  |  |
| - T3 | 3 | 3 |
| Resection status |  |  |
| R0 | 2 | 3 |
| R1 | 1 | 0 |
| Pathological response (CRT^+^ group) |  |  |
| Complete response | 1 | 0 |
| Major pathological response | 2 | 1 |
| Residual viable tumor > 10% | 0 | 2 |
| Median radiation total dose (Gy) | 48 (46 – 48) | 46 (44 – 50) |
| Median radiation dose per fraction (Gy) | 2 | 2 |
| Radiation technique |  |  |
| Conformal 3D RT | 1 | 0 |
| IMRT | 2 | 3 |
| Number of concurrent chemo cures | 3 | 3 |
| Mean time interval between end of radiotherapy and surgery (days) | 127 (87 – 198) | 97 (71 – 131) |
| Year of treatement | 2010 ; 2014 ; 2016 | 2017 ; 2019 ; 2019 |
